# Editorial Notes

**Published:** 1933

**Authors:** 


### Editorial Notes

**Dr. Symonds and Cholera in Bristol.**

A MANUSCRIPT volume has recently been presented to the University Medical Library by Dr. A. G. Gibson of Oxford, who stated that it was given to him by the late Horatio Symonds, a well-known practitioner in Oxford and nephew of our Dr. Addington Symonds of the General Hospital. Dr. G. Parker, our Honorary Medical Librarian, has examined the manuscript, and makes the following comments upon it:—

The greater part of the book is taken up with case histories from 1832 to 1836 of patients, most of them living in Bristol, *e.g.* W. Burke, staying at the Rummer Hotel after crossing from Dublin, August 1835. At one end there is a "Monthly confidential return of cholera cases in District No. 3 West of the City and County of Bristol." As a matter of fact, this "Monthly" return is continued on and on from 19th August to 6th November, and includes 107 cases in the Lewins Mead district.

After looking over the clinical notes one comes to the conclusion that, though the writing differs very much in places, it is all by the same hand that signs a loose prescription for Lydia Hurley, one of the cases. The signature is undoubtedly by John Addington Symonds, and this explains the presence of an official return of cholera cases. Symonds had not long been in Bristol when the great cholera outbreak of 1832 took place. He was made one of the Commissioners of Health in July, and became Hon. Secretary to the Board a month or so later, and he signs henceforward the monthly returns for the whole city. Some of these returns with his signature can still be seen in Richard Smith's M.S. Collections at the Bristol Royal Infirmary, vol. 1828-36. The return we have here has names, previous health, etc., of all patients in a district, evidently not for publication, but the basis on which the returns for the whole city were made out and sanitary measures devised.

J. A. Symonds also wrote an able history of this epidemic and published it three years later in the *Transactions of the Provincial Medical and Surgical Association*, vol. iii., p. 170. There he tells us that the main outbreak began on July 11th, with a case in Harford's Court on the bank of the Frome, to be followed by others in the prison on the New Cut, Redcliffe, the Temple and St. Augustine's. St. Philip's and Bedminster were also severely visited, and worst of all, the over-crowded poor-house, St. Peter's Hospital, in which 208 cases occurred. The total cases reported amounted to 1,612, and the deaths to 626. He discusses the possible means by which infection could be transmitted, but curiously enough omits the possibility of water carriage. He found the mortality the same in both sexes, and greatest above the age of fifty. It is curious that while not a single doctor was attacked, almost every nurse and stretcher-bearer went down with it. This epidemic was twice as large as the one in 1849, in which the heroic work of Gage Parsons and Williams took place, as recorded on their monuments in Arno's Vale.

One is apt to think of Symonds only as a very successful and fashionable consultant, with marked literary and classical tastes, but this note-book shows him, four or five years after qualification, working hard at the clinical and sanitary studies which were the basis of his mental equipment.

**Burden Mental Research Trust.**

IN January last Mrs. R. G. Burden, Warden of the National Institutions for Persons Requiring Care and Control, announced through the medium of the Council of the British Medical Association her offer of the sum of £10,000 for the encouragement and prosecution of research into mental problems and disorders.

An Advisory Committee has been formed consisting of the following members: Professor R. J. A. Berry, Director of Medical Services, Stoke Park Colony

(Chairman), Mrs. R. G. Burden, Professor E. D. Adrian, Sir Henry Brackenbury, Miss Ruth Darwin, Miss E. M. Elderton, Dr. R. A. Fisher, Miss Evelyn Fox, Dr. E. O. Lewis, Dr. W. Rees Thomas, Mr. L. G. Brock and Dr. G. C. Anderson (Honorary Secretary and Treasurer).

It will be seen that this Committee is thoroughly representative of national, medical and scientific interests; its meetings will be held in London at the House of the British Medical Association, whilst the headquarters of the research will be the laboratories at Stoke Park.

The Committee visualizes a five years' organized period of research, and has already invited applications for the position of Principal Investigator, with whom, after appointment, the Committee will discuss and formulate its further plans. Other appointments, including those of Social workers, will be advertised and made later.

Whilst the exact scope of the research remains to be defined there is no doubt of its urgency, and the munificent gift of Mrs. Burden will be gratefully acknowledged far and wide, but nowhere with greater appreciation than in Bristol, where the scientific headquarters of the research are to be placed.

**Death of Sir Percy Sargent.**

A VERY loyal Bristolian and a most eminent neurological surgeon has passed away in Sir Percy Sargent. He was born in Pembroke Road and educated at Clifton College. His medical studies were conducted at Cambridge and St. Thomas's Hospital, where he became Senior Surgeon. At the time of his death he was a member of the Council of Clifton College and represented the Royal College of Surgeons on the Court of Bristol University. His wife was a daughter of the late Sir Herbert Ashman and had predeceased him by a few months. Sir Percy Sargent was a keen Freemason and rose to high distinction in the craft.

Sargent was a man of whom Bristol may well be as proud as he was proud of his birth-place. Quiet, courteous and capable, he held his place in the estimation of his friends, his patients and his colleagues by affection even more than by his outstanding talents.

